# Artificial consciousness: the missing ingredient for ethical AI?

**DOI:** 10.3389/frobt.2023.1270460

**Published:** 2023-11-21

**Authors:** Antonio Chella

**Affiliations:** RoboticsLab, Department of Engineering, Università degli Studi di Palermo, Italy & ICAR-CNR, Palermo, Italy

**Keywords:** artificial consciousness, robot ethics framework, ethical AI, robot consciousness, cognitive architectures

## Abstract

Can we conceive machines that can formulate autonomous intentions and make conscious decisions? If so, how would this ability affect their ethical behavior? Some case studies help us understand how advances in understanding artificial consciousness can contribute to creating ethical AI systems.

## Introduction

In April 2023, the prestigious Association for Mathematical Consciousness Science (AMCS), which brings together researchers studying the theoretical aspects of consciousness, published an open letter entitled “The Responsible Development of AI Agenda Needs to Include Consciousness Research^1^.”

This letter came in response to the Future of Life Institute’s letter regarding the proposed moratorium of at least 6 months for training AI systems of the GPT-4 type^2^. The letter, whose signatories include distinguished Turing Award scholars such as Manuel Blum and Yoshua Bengio, and many other scholars active in AI and consciousness, calls for research on AI to be coupled with consciousness research.

In Chella et al. (2022), some key theoretical aspects of artificial consciousness studies are reviewed, introducing the main concepts, theories, and issues related to this field of research.

Two recent review papers, by Chalmers and by Butlin et al., summarize the state-of-the-art of artificial consciousness. Chalmers (2023) analyzes the possibility that a large language model, such as ChatGPT, may eventually be conscious by reviewing some commonly accepted indicators for consciousness. Examples are the capability of self-reporting and seeming conscious and conversational, as well as general intelligence capability. Chalmers also analyzes structural capabilities, such as the presence of senses and embodiment, the capability of recurrent processing and building a model of self and the environment, and the presence of a global workspace and unified agency. Chalmers then rules out the possibility of artificial consciousness in the current version of ChatGPT because it lacks all these capabilities.

A similar strategy is taken by Butlin et al. (2023). The authors consider the prominent theories of consciousness in the literature: the recurrent processing theory, the global workspace theory, the higher-order theory, the attention schema theory, the predictive processing, and agency and embodiment capabilities. Then, the authors outline the indicator properties derived from each of these theories. Considering these indicator properties, the authors conclude that no current AI system is a strong candidate for consciousness.

This mini-review emphasizes the crucial importance of artificial consciousness studies in creating ethical AI systems—notably, both papers by Chalmers and Butlin et al. emphasize the ethical challenges associated with artificial consciousness.

The debate is specifically about whether or not a moral agent requires a form of consciousness to act ethically. This issue has generated intense debate within the scientific community, with theorists taking opposing positions and some favoring that consciousness is a necessary component of ethical behavior. In contrast, others believe it is not essential. See, e.g., Levy (2014) for a summary of the various philosophical positions.

More clearly, this mini-review hypothesizes that, in principle, an AI system, when equipped with some form of artificial consciousness, may act as a moral agent. This hypothesis is debatable, and researchers in robotics may disagree. For example, Wortham (2020), in investigating robot architectures, clearly states that moral agency is an attribute of humans. Moreover, he shows that simple and transparent robot architectures may implement aspects typical of moral agency, such as the capability of selecting actions. Bryson (2018) argues that while it may be, in principle, possible to build an AI system acting as a moral agent or patient, it is not necessary or desirable. According to Bryson, “robots should be slaves” (Bryson and Wilks, 2010).

At the heart of this debate is the fundamental question regarding the “capacity to have intentions” and whether this capacity can be extended to machines. In other words, can we conceive machines capable of formulating autonomous intentions and making conscious decisions? If so, how would this ability affect their ethical behavior? In this short review, we will explore these issues in more depth. We will analyze some case studies and computational theories and discuss how advances in understanding artificial consciousness may contribute to creating more ethical AI systems. We will provide an up-to-date overview of current positions in this field, emphasizing the challenges and opportunities ahead as we attempt to develop machines with a form of ethics.

## Case studies of ethical AI systems inspired by artificial consciousness

The definition of Artificial Moral Agent (AMA) was introduced by Wallach and Allen (2009). Wallach and Allen analyze two specific characteristics of AI systems: their autonomy and their ethical sensitivity. The authors divide their operation into three categories. The first category concerns AI systems for which morality is merely another operation; these systems are typically marked by low autonomy and ethical sensitivity. The second category concerns systems with moral functionality. These systems exhibit medium autonomy, where ethical sensitivity is present at the functional level. Finally, the third category concerns systems with high autonomy in which ethical sensitivity is inherent in the system itself.

According to Wallach and Allen, current AI systems are all marked by medium to high autonomy but low ethical sensitivity and are a potentially high risk to humanity.

## Top-down systems

Approaches toward ethical AI systems are typically based on top-down, bottom-up, and hybrid approaches. Arkin (2009) introduces and discusses several examples of top-down systems. The basic idea is to have a robotic system governed by an AI architecture in which the rules of engagement, just war rules, the UN Declaration of Human Rights, the Geneva Convention, etc., are implemented. Then, before executing any action, the AI system verifies that it is compatible with all the implemented rules and constraints.

Arkin’s motivation is to ensure that the actions of AI systems always adhere to ethical rules. However, Arkin’s proposed ethical systems need to consider that the rules, which are universally agreed upon, may need to be revised for a machine to interpret in practical cases. Take, for example, the well-known three laws of robotics proposed by science fiction writer Isaac Asimov. Although these laws are sharable, their interpretation can lead to ambiguities, and, in fact, much of Asimov’s robotics stories arise from ambiguities in interpreting these laws.

## Global workspace theory

Wallach et al. (2011) proposed an architecture for an AI system that intends to overcome the limitation of Arkin’s top-down approach. Their proposed system is based on the Global Workspace Theory (GWT) originally proposed by Baars (1997), which is, to date, one of the most widely followed theories in the field of consciousness studies. In addition, there are several implementations of it (Signa et al., 2021).

In short, following GWT, the brain can be functionally considered as a set of specialized, unconscious processors. On the other hand, consciousness acts serially and with limited capacity and is associated with a global workspace. Unconscious processors work in parallel and compete for access to the global workspace. When a processor wins the competition, it accesses the workspace and, through it, sends its contents to other processors to recruit them. The conscious event is generated by the processor that wins the competition and takes control of the workspace. This architecture has been analyzed from the perspective of creating an ethical AI system because it allows for a hybrid approach. In the case of an AI system, the various unconscious processors carry out moral analysis of a problem from different perspectives, such as from the point of view of deontological and utilitarian aspects, the analysis of values involved, prior experience, and so on. The different processors then compete for control of the workspace. When one processor, corresponding to a specific point of view, prevails, it takes control of the workspace and generates the appropriate action.

Thus, a GWT-based AI system is a more versatile agent than the top-down systems hypothesized by Arkin and could adapt to different ethical situations with different viewpoints and experience levels.

The previously cited moral philosopher Levy (2014) analyzed GWT from an ethical perspective as a model of human consciousness. He concludes that an agent is responsible for his or her actions only when GWT is fully operational. Only then does the agent want to perform that action and can thus be held responsible since the relevant unconscious processor that generated the action has effectively controlled the GWT. Levy analyzes anomalous situations in which some subjects performed actions in situations of altered consciousness. In these cases, a processor takes control of the actions without going through GWT. Levy hypothesizes that, in these situations, the subject may not be held fully responsible for his or her actions.

Levy does not refer to AI systems, but his considerations can also be extended to AI systems. Thus, it is possible to assume that an AI system is responsible for its actions when it has a GWT and chooses actions based on a fully operational GWT.

Along these lines of thought, Bridewall and Bello developed the ARCADIA software system (Bridewell and Bello, 2016), which takes its cue from GWT and implements its focus of attention mechanism. According to the authors, and in agreement with what Levy discussed, a machine can be considered ideally responsible for an action only when that action is chosen by committing all computational resources.

Bello and Bridewell (2020) then simulated a situation in which the ARCADIA system, driving a car, hits a pedestrian as the pedestrian crosses the road. In one scenario, the system’s focus of attention points to the center of the roadway; the car has a straight trajectory following the road, and the pedestrian enters from the left into the system’s focus of attention. In this case, the accident, according to Bello and Bridewall, was not voluntarily caused by the system.

In the second scenario, however, the focus of the system’s attention is caught by the pedestrian on the left, and the system precisely corrects the car’s trajectory to center the pedestrian. In this second case, the system has thus used all computational resources to account for the pedestrian and can, therefore, be held responsible for the pedestrian’s involvement.

## Internal models

An AI system inspired by artificial consciousness and based on a different approach was proposed by Winfield and Pitt (2014). The idea on which Winfield’s system is based is inspired by the theory of internal models of consciousness. According to this theory [see, e.g., Hesslow (2002) and Holland (2003)], the mind constructs an internal model of itself, including its own body, and a model of the external world. Conscious interaction occurs within the mind, between the model of one’s body and the external world.

This theory has the merit of justifying the mental imagery and simulative capabilities of the mind. Implemented in an autonomous agent, it requires the agent to have the ability to reconstruct a model of itself and a model of the external world.

According to the system proposed by Winfield, the robot builds a simulation of the world in which it can simulate its movements. Therefore, when the robot perceives a person walking toward a dangerous place, e.g., a ditch, it can simulate the optimal sequence of actions to prevent the person from falling into the ditch by interposing between the person and the ditch.

From these considerations, Vanderelst and Winfield (2018) describe a complex architecture for controlling an ethical robot. This architecture contains an internal model of the robot, a model of the external world, and a limited model of human behavior. The system can generate plans and make ethical evaluations of the plans generated. The weakness of this approach is the need to create a model of the robot and a model of the external world. However, extensive progress has been made in these directions: Lipson’s group recently developed an algorithm that allows a mechanical arm to build a 3-D model of itself from images taken by external cameras, as if the robot were looking in the mirror (Chella et al., 2020). Extensive progress has also been made in the 3D reconstruction of environments from images, thanks partly to recent advances in deep learning (Han et al., 2021).

## Artificial empathy

An interesting strand of research hypothesizes that a robot can only behave ethically toward people if it can empathize with them. Empathy is thus the basis of proto-morality.

Asada (2020) proposed a complex architecture that takes cues from the neuroscience of pain and relief to simulate artificial empathy. Specifically, Asada incorporated a pain-related nervous system model into a robot to simulate the feeling of pain. In addition, by simulating a mirror neuron system, the robot can develop a kind of emotional contagion, and thus, empathy.

According to Metzinger (2021), the study of artificial consciousness should be subject to a moratorium until 2050 because a machine with an artificial consciousness might be able to suffer.

From a positive point of view, Metzinger, and Agarwal and Edelman (Agarwal, 2020), have debated the possibility of constructing an artificial system endowed with consciousness but without suffering. In summary, according to these analyses, a system endowed with artificial consciousness could limit suffering through experiences reminiscent of meditative states typical of the Buddhist tradition.

According to Man and Damasio (2019), under certain conditions, machines capable of implementing homeostatic processes could acquire a source of motivation and a means of evaluating their behavior, similar to feelings in living organisms. Technically, Man and Damasio analyze homeostatic systems based on reinforcement learning, such as those described by Keramati and Gutkin (2014). In this way, a robotic system might be able to associate a perturbation of its homeostatic state with a feeling. A perturbation that moves the robot away from its stable homeostatic state might be associated with a negative feeling. In contrast, a perturbation that brings the robot closer to its stable homeostatic state might correspond to a positive feeling. In this way, the robot, being able to feel something like a feeling, could also feel some empathy for people and possibly other robots.

## Self-organizing dynamics

Tani (2017) discussed a model named MTRNN (multiple-timescale recurrent neural network) based on a hierarchy of fully connected recurrent networks controlling a robot. Fast time constraints characterize the networks at the lower levels of the hierarchy and are related to processing information from robot sensors and generating robot movements. The networks at the intermediate levels, characterized by intermediate time constraints, are related to the generation and processing of sensory and motion primitives. In contrast, slow time constraints characterize the networks at the higher levels of the hierarchy and are related to the recognition and generation of action plans.

Then, MTRNN operation is characterized by self-organization of the hierarchy consisting of the bottom-up acquisition of sensory data and the top-down generation of action plans related to the robot’s intentions, which in turn trigger sequences of behavior primitives and movements. Tani showed that a sort of “free will” may be observed in the architecture when the higher-level networks spontaneously generate the robot’s intentions through chaos. Then, when a gap emerges between the top-down generated intentions and the bottom-up perception of the external world, conscious awareness of intentions arises to minimize this gap [see Tani (2017), Chap. 10].

Tani disputes that this mechanism of free will may allow the robot to generate either good or bad behaviors. However, the robot may learn moral values such as its behavior. Then, it may learn to generate good behaviors according to its values and to inhibit bad behaviors.

## Cognitive consciousness

A completely different approach from the one described above was proposed by Bringsjord and Naveen Sundar (2020). The authors axiomatically define “cognitive consciousness” as the functional requirements that an entity with consciousness must have, without regard to whether the entity feels anything. The authors then define a cognitive logic that roughly coincides with a family of higher-order quantified multi-operator modal logics for formally reasoning about the properties of consciousness. The characteristics of an entity endowed with consciousness are then formally defined through a system of axioms. The authors also implemented an automatic reasoning system and a planner related to systems endowed with consciousness.

An interesting aspect of the theory concerns the definition of a measure, called Lambda, the degree of cognitive consciousness of an entity. The Lambda measure provides the degree of cognitive consciousness of an agent at a given time and over intervals composed of such times. The measure has interesting aspects: it predicts null consciousness for some animals and machines, and a discontinuity in the level of consciousness between humans and machines and between humans and humans. One debated aspect concerns the null consciousness prediction for AI agents whose behavior is based on learning about neural networks.

Naveen Sundar and Bringsjord (2017) also built an AI system capable of reasoning about the doctrine of double effect and the well-known trolley problem and measured its level of consciousness. It follows from this study that reasoning about the doctrine of double effect requires a fairly high level of cognitive consciousness, which is not attainable by simple AI systems.

## Artificial wisdom

“Artificial Phronesis” or artificial wisdom considers an artificial agent who is not bound to follow a specific ethical theory, such as the double-effect theory or the deontological theory, but possesses the general ability to solve ethical problems wisely (Sullins et al., 2021).

According to this approach, an ethical agent should perform his or her actions based on wisdom and not through mere implementation of ethical doctrines. Following Aristotle, the ability to act wisely cannot be formalized through rules but is a practice that the agent must acquire through experience. Real situations are generally complex; each is encountered for the first time and thus lacks prior experience. Artificial wisdom, therefore, requires a wise agent to have the ability to understand the context, that is, what the actors are and what is at stake. The agent must also have the ability to learn new contexts and improvise on predefined patterns; it must be aware of the actions and potential reactions of other actors.

Finally, the agent must be able to revise its behavior by analyzing the interactions made. An early implementation of an agent based on artificial wisdom was described by Stenseke (2021).

In this vein, Chella et al. (2020) and Chella et al. (2024) are studying the effect of robots’ inner speech on artificial wisdom. Specifically, the research has focused on experiments in which a user and a robot must perform a collaborative task, such as setting a dining table in a nursing home where people with dementia are also present. The experiments analyze how a user, by hearing the robot’s inner speech during the collaborative task, can achieve a higher degree of awareness of issues related to people with dementia. Preliminary results support this hypothesis.

## Conclusion

In this mini-review, we analyzed case studies focused on ethical AI agents inspired and influenced by various theories of artificial consciousness. This process allowed us to critically explore different facets of this complex topic.

Two of the most challenging questions concern whether an AI system may be a moral agent and if a form of artificial consciousness is needed to ensure ethical behavior in the AI system. These questions have no definitive answers and remain essential open lines of research. The problematic nature of the issue lies in defining what we mean by “consciousness” in a non-biological entity and in delineating the criteria to measure the ethics of an action performed by an AI system.

Finally, we mentioned another major open issue: the importance of research on consciousness and emotion studies in machines for progress toward more ethical AI.

This debate reflects a broader and more fundamental issue: the ability of machines to “feel” or “understand” authentically and how that ability might influence their ethical behavior.

These issues are dense with theoretical, methodological, and ethical implications and challenges that the scientific community cannot ignore. Their complexity is a reminder of the importance of a multidisciplinary approach in AI research, combining computer science, philosophy, psychology, neuroscience, and ethics to develop AI systems that are not only technically advanced but also ethically responsible.

## Footnotes

1
https://amcs-community.org/open-letters/

2
https://futureoflife.org/open-letter/pause-giant-ai-experiments/
